# American Academy of Dental Science

**Published:** 1899-08

**Authors:** 

**Affiliations:** American Academy of Dental Science


### Reports of Society Meetings.

### AMERICAN ACADEMY OF DENTAL SCIENCE.

The regular monthly meeting of the American Academy of Dental Science was held at Young's Hotel, Wednesday evening, February 1, 1899, at six o'clock.

A paper was read by Frederick T. Rogers, M.D., of Providence, R. I. Subject: "The Ethical Advertiser."

(For Dr. Rogers's paper, see page 504.)

*Dr. Rogers.*—I take it, gentlemen, no apology is needed for presenting to you to-night this paper, which was originally written for a medical society. It was written to be read before the American Academy of Medicine at Denver, last June, but as I look at the field which lies before all medical men the abuse to which I desire to call your attention is much the same in your profession as in mine. The man who advertises in the paper that he "cures fits" is in the same category as the man who plasters the front of his office with the sign "Painless Extracting." The man who rushes into print with a peculiar case is doing something which is obnoxious to the better element of the profession to which he belongs, whether it be medicine, dentistry, or any of the learned professions.

### DISCUSSION.

*Dr. Bradley.*—It was with some hesitation that I consented to open this discussion when the chairman of the Executive Committee invited me to do so, feeling that my experience was of a limited nature, and I was certainly not an authority upon the ethics of the profession; in fact, I have given but very little attention to this subject and have thought very little about it, except when some noticeable violation of it has come to my notice. However, I may act as a wedge, perhaps, this evening, and others who will wield the sledge will either drive that wedge in, or if it does not suit them, substitute another which will accomplish the purpose of provoking a general discussion.

I think, Mr. President, there is room for considerable satisfaction to the members of the Academy in having a special paper read before us by a member of the medical profession on a subject which so vitally interests all professions. Not many years ago, I presume, it would have been unusual for medical men to read their papers before a body of dentists, even before an organization of such recognized standing as the American Academy of Dental Science, and the fact that this has been done this evening by a member of the medical profession, and a member of that important body, the American Academy of Medicine, indicates that we have a standing with our medical brothers, that we are recognized as a part of those who are engaged in the healing art, so I feel that we may be pardoned if we congratulate ourselves that our position has come to be openly recognized.

The question of the code of ethics is something that has always been a vexed one as regards its interpretation. Our own Code of Ethics does not speak at very great length about advertising. I believe the only place where anything is said is in Section 4, Article II., of the Code, which reads as follows:

"It is unprofessional to resort to public advertisements, cards, hand-bills, posters, or signs, calling attention to peculiar styles of work, lowness of prices, or special modes of operating; or to claim superiority over neighboring practitioners; to publish reports of cases or certificates in public prints; to go from house to house to solicit or perform operations; to circulate or recommend nostrums, or to perform any similar acts."

I think this article, though not very long, covers the ground quite thoroughly. It speaks in a guarded manner about specialism in the profession and of advertising in the public press or by using posters. It also speaks in a guarded manner of the ethical advertising by the publishing of reports of unusual cases in the public prints, which the essayist has brought out so forcibly.

It seems to me that the dental profession has a little different stand-point from the medical profession on this question. I doubt if very many of our members really engage in specialism as is done in the medical fraternity. We may have one or two instances, perhaps, but I am not aware of very many who give their whole attention to one phase of the work, so that we shall have to look at our advertising a little differently from what the essayist has done. I feel with him that the profession of dentistry is somewhat crowded by those who have entered merely from the mercantile point of view. There are too few educated men, or those who are gentlemen by culture or instinct, and they look at it too much from the standpoint of keen competition; they try to feel that it is legitimate to take patients from any source that they can; but I am pleased to think that a very large proportion—and I feel sure that the members of the American Academy of Dental Science, as well as of many other dental societies—may be classed as those who have entered the profession feeling that the claims of their fellow-practitioners have always to be carefully considered.

In the matter of advertising in the daily press, I was talking with the proprietor of a daily paper, and he said that, while the medical men and dentists never advertised in the papers, he had observed they were always very glad to have a notice in the paper of what they did, and that was really more valuable to them than half a column of advertising. I have faith to believe, Mr. President, if I can judge from my acquaintance, that a very great many of my profession and of the medical profession really desire not to see their name in the public press; they take particular pains not to have it appear, even in social and political affairs as well as in professional matters. I must claim that the reporter, the ubiquitous reporter, the ever-present reporter, is responsible for the advertising which the professional man many times wishes that he did not get. I know of a case of a friend of mine who went on a little vacation to the White Mountains, and on his return brought a few plants with him, and a notice appeared in the paper something like this: "Dr. ——— has been spending some time in the White Mountains, and has brought home some plants and has established quite a garden." Now, I know that it was very distasteful to the gentleman to have that appear in the paper.

Another way, I think, in which these notices come before the public is by persons soliciting testimonials from professional people regarding something they use which may have more or less merit. Within a year I have had, and I presume most of you have had, the same experience, of some one calling on you and asking you to become stockholders in a concern manufacturing some drugs, and promising great returns from whatever capital you invested, and to prove that everything was all right he showed a long list of people who had agreed to take stock in this concern. In other cases you receive a letter from some firm manufacturing a tooth-powder or tooth-wash, in which they state that if you will prepare an article on the proper use of the brush and the hygiene of the mouth, they will have a pamphlet printed with your name on it, if you wish, and will send you any number of copies that you want, or, if you will mail them a list, they will send them to any addresses you suggest, thus serving the double purpose of advertising both for the dentist and the manufacturer. Letters of this kind—and I have had a number of them—I have paid very little attention to, and throw them at once into the waste-basket. But it seems to me that the profession is not entirely to blame if they fall into these little traps. Very frequently it is those who are catering to the profession from a mercenary stand-point who will lay things before the members of the profession in such a manner that they do not appear to violate the code of ethics. I have come to think that there is a great deal in the rule laid down by Robert Burns, that before doing anything about which there is a doubt, one should think how it would look in the other fellow to do it. If we think it would be unbecoming for A or B to do thus and so, to bring his name before the public in certain ways, or to lend his assistance to unprofessional schemes, we better decline to be associated with those things ourselves.

In regard to the question of professors and instructors in dental schools occupying positions similar to the surgeons and physicians to hospitals, and whether it is correct for titles of that kind to appear in connection with the name of such a person, I know that the schools issue prospectuses and announcements, in which attention is called to the prominence, the reputation, and the skill of the instructors. These announcements appear in the professional journals, which are circulated largely among the profession, and, perhaps, are seen somewhat by the laity, and while the essayist claims that such announcement of the degrees that a professor may hold or mention of the work that he is in charge of at the school is in a certain sense ethical advertising, yet, have we not a duty to the schools? Let us look at it from that point of view. The public desires to know who are the teachers and the professors having in charge the education of those who wish to become members of the profession. It is not sufficient to state in the announcement, "We have a good corps of professors and instructors;" the public must know who they are, and while it may advertise the instructors, the professors, and the clinical demonstrators, and bring them before the public as especially skilled in certain lines, still it is for the benefit of the school, it is for the benefit of the educational plant, if we may say so, of the work we are engaged in, that the students may know in whose hands they shall intrust their education. So, it seems to me, there are two sides to this question, and that while the mention of the hospital assignments against the names of the faculty in the announcements of dental colleges does advertise those officers to some extent, there is something to be said in favor of building up the school by the reputation of the faculty, and insuring its students the best dental education.

In regard to formulating a law to regulate this ethical advertising. I question if any one would like to undertake it, as it it would certainly be very difficult to do. It seems to me, after thinking the matter over, that an unwritten law is the best. Sometimes there is more strength in an unwritten law of this character than there would be in one that is formulated; but let us have this feeling among members of the profession: that we are to be gentlemen; that we are to consider the other man. Some one has said that one of the grandest works that any one can find to do is to be engaged in discovering the other man, and I think if we turn our attention to discovering what we can do in this line there will be no need of formulating laws with regard to "ethical advertising."

*President Cooke.*—I am sure this is a question in which we are all interested and about which a great deal may be said. There is rather a good joke on myself in this connection which I at once appreciated. When I received my sheep-skin from the school, I thought it would be the proper thing to have it properly placed, so I had it framed and hung it up on the wall, and thought no more about it. A few years ago I had a small boy in my chair who was fully up to the average in inquisitiveness, and, pointing to the diploma, he asked, "What's that for,—to advertise?" I said, "Yes." Nothing more was said, for I thought the boy's intuition had got the thing nearly right, and it was hardly necessary to say anything more, as it did appear to be one form of advertising.

One evening I had a little time to spare and was walking down Washington Street, and I came in front of one of those glass cases in which there were some fine pieces of crown- and bridge-work, and I stopped for a moment to look at them, and as I did so I heard a voice at my elbow, "Examination free, sir, walk right up." I turned and saw there a colored gentleman, in a uniform, with large brass buttons, looking very gorgeous, and the inducements to have your dental work done there seemed to be very attractive, but I did not walk up. Now that is the other form of advertising.

I am sure we are all interested in this subject, and the one little article which to my mind covers this point in our By-Laws is Section 1 of Article II.

"In his relation with another dental practitioner and his patients, the dentist should be governed by strict rules of honor and courtesy. His conduct should be such as, if universally imitated, would insure the mutual confidence of all dental practitioners."

That seems to me to express the whole thing in a nut-shell. If a man would say to himself, This action of mine, suppose everybody else did it, what sort of a profession, what sort of a society would we have? Suppose Dr. So-and-So did the same thing to my patient that I propose doing to his, how would I like it? By asking that question of ourselves we could very easily determine whether the act was professional or not. If we act as we should like to be treated under similar circumstances, we shall not have much trouble with the subject of ethical or non-ethical advertising.

*Dr. Briggs.*—I thought while the essayist was reading we all felt that we knew some cases in ourselves or in others that we thought steered pretty close to some form of advertising that was open to criticism. It is a very vexed question,—what we shall let the other fellow do in the way of advertising. The legitimate notice that the country practitioner puts in the local paper is the one thing that is frowned upon most, as the essayist has brought to our notice. At the same time, we can understand how that might be proper and decorous, while the ways which the city practitioner employs to bring himself into prominence, although within ethical bounds, may be most flagrant examples of advertising. I do not know how we are to treat this subject. It is difficult to distinguish between that which is done directly and that which comes about indirectly without the knowledge or consent of the person who is being advertised. The public prints are taken out of the hands of the practitioner, and in one sense you might say that some of the operations performed become things of public interest to the extent that they are discussed and commented upon by the laity and the lay press. Take, for instance, a case which recently appeared, which on the first blush seemed to be a most flagrant case of ethical advertising; the advertisement, if you might call it that, of the operation of the removal of a woman's stomach. Now, that is an extraordinary operation; an operation which is undoubtedly of a great deal of interest to the laity as well as the members of the surgical and medical profession, and the surgeon who was mentioned there probably needs advertising as little as any surgeon in this city, and yet those of us who read it regarded it as something of an advertisement, and we had the feeling that if we were to furnish the details of our peculiar cases to the public press that it would be so regarded; but, on the other hand, we rarely have things come into our practice that are of equal public interest.

Then there are men who do advertise themselves quite as effectually in their own offices without going outside into the public prints. You cannot reach these people by formulating laws, and the only remedy that suggests itself to me for getting at this matter for treatment is the one mentioned by Dr. Bradley,—that of educating the young men in the schools up to a high standard of moral decency and of genteel deportment, as it were, and to depend upon that to bring out the right sort of conduct towards his fellow-men.

*Dr. Andrews.*—I think Dr. Briggs hit the nail squarely on the head when he spoke of educating our students in ethics at the time they are being taught in our schools. I have had occasion to state to people who come to me soliciting advertising cards for charitable programmes, that it is something I never do; when they are persistent, I tell them that if they will get Dr. So-and-So's card, I will consider the matter. They say, "Oh, he is a physician, and physicians do not advertise. They do not think it professional." I tell them that I see no reason why the same rules should not govern the dentist, and that ends the matter. There are various ways in which a name may be brought to the attention of the public, and I suppose it is all right, perhaps, but, as Dr. Briggs says, if we educate students in the schools on the subject of their conduct towards other members of the profession, we should probably have less to discuss on this subject.

*Dr. Wilson.*—I believe thoroughly in the idea that our young men should be educated as gentlemen, but experience has shown us in the past that there is a great diversity of opinion even among gentlemen about various matters pertaining to our profession. Some years ago, some of our members committed certain indiscretions, which, in the opinion of the majority, were considered unprofessional. This was during a time when we had no code of ethics, and the result was a series of discussions and wearisome talks, which need never have taken place if we had been properly armed and equipped with a code of ethics. This being the case, it seemed to me essentially necessary that we should have a code of ethics, so that all present members, and those to come, might know just what the Academy expected of them. Did I understand Dr. Bradley to say the section he read was not quite complete?

*Dr. Bradley.*—I said that that was all that it contained in reference to advertising, ethical or otherwise.

*Dr. Wilson.*—I want to say that that section is an exact copy of the Code of the Massachusetts Medical Society. The only difference being in the substitution of the word dentist wherever necessary. The article which Dr. Cooke read was also taken from the Massachusetts Medical Society. It seemed to me better to take the best there was from the various prominent medical and dental societies in the country,—codes which had stood the test of time, and seemed to be in every way satisfactory, rather than originate any new ideas. The result was the adoption of the present Code of Ethics.

*Dr. Smith.*—Dr. Briggs refers to a case which he said looked like a flagrant case of advertising. True, it does, but no one who knows that surgeon believes for a moment that he had anything to do with it. The case came to him in the course of practice, and he believed that was the best method of dealing with it: the reporter got hold of it, wrote about it, and in that way the operation was brought before the public, and the feeling of opposition to publicity by the operator was not considered. Now, if a reporter happens to get hold of a few facts about this meeting we may read in the paper to-morrow morning that Dr. Rogers, an eminent specialist, of Providence, gave a very instructive and entertaining discourse before the American Academy of Dental Science last evening. It may be that he would prefer not to be so spoken of, but what could he do?

There has always been considerable discussion regarding the right of the learned professions to advertise. When we speak of learned professions, we are generally understood to mean divinity, law, and medicine. Now, if it is unprofessional for members of the medical profession to advertise, certainly it is unprofessional for the members of the professions of divinity or of law to advertise, but I know of no class of men whose names appear in the newspapers oftener than do those of the professions of divinity and law. What is the result? The public knows more about the members of those professions and respect them accordingly. They know more about medicine to the extent that it is advertised in a legitimate way. The newspaper is the medium through which the public learns what is being done by different people all over the world, and they have a right to print anything which is legal that they think will interest their readers. Referring again to the case which was cited this evening, the account of the removal of a woman's stomach. As I have said, the surgeon did not need the advertising which he received through the publication of that article, but was not an operation of such a character of great interest to the public? In all probability nearly every person who read that item of news was surprised to find that such an operation was possible and the patient live, and naturally they would have a very high estimation of the man who was skilful enough to accomplish it. Not only that, but had not the public a more exalted opinion of the whole medical profession, and in that way every member of it came in for a share of the benefit of that advertisement?

I was much impressed one evening by a remark of Rev. Dr. McKenzie, in speaking about the dental profession, when he said that we might be called the "silent profession;" that the public really knew little about us, about what we have done, or what it was possible for us to do. And so, while our praises are in the mouths of our patients, many times they get no further than that; and many of them, I think, are terribly silent, and so we suffer in that sense from a lack of advertising which would reasonably seem to belong to us.

In regard to these show cases, etc., my position has brought to me different phases of this question and its results. To a young man who has prepared himself for a profession and is fortunate enough to drop into an established office with a man who has a large practice, he never need resort to a card or the newspaper to advertise himself; but where there is one young man so situated, there are hundreds and thousands of young men who have to begin differently; many of them are poor and, perhaps, have borrowed money in order to obtain their education, and are struggling to pay back the loan. They have high ethical ideas of what is right, and those high ethical ideas lead them to start in a prominent and cultivated part of the city, but the hoped-for practice does not come, and hunger and want soon drives those ethical feelings out of the best of men. I have had those men come to me and say, "Dr. Smith, I must give up my office; I am not paying expenses where I am, and I must go down to such and such a part of the city, and see if I cannot make a living there." I tell them that it is just as honorable to practise in that part of the city as any other, provided the practice is respectfully conducted. So they change their office and start again, using the same signs. But they find they do not get patients, and they think they must get a larger sign, or perhaps several of them, to attract the attention of the people, and it is a question in my mind whether a person in that situation does not have to do something of that kind to call the attention of the people to him. With the other man, it is not necessary to do anything of that kind; he has been introduced, and his praises have been sounded from patient to patient, and he is thus advertised,—of course, in a proper way, and soon has a lucrative practice.

*Dr. Williams.*—I remember several years ago when the subject of medical legislation was being discussed in the State committee, I was talking with John I. Baker, a veteran legislator, and in the course of the conversation he made this remark: "You cannot by legislation make men conscientious," which seems to harmonize with the ideas which Dr. Bradley and Dr. Andrews hold on the subject, and also to be in the same line with the remark of Dr. Henry J. Bigelow, when the subject was being discussed in the Massachusetts Medical Society, which was to the effect that you may make all the laws you please about ethics, but there are people who will get around them.

This matter of necessity is a difficult thing to deal with. It makes the man who is under obligation to support himself, and perhaps a family, conspicuous, from the fact that he makes the attempt to bring himself before the public on the same line as the "milking stool" man, or the show-case man that has been referred to. But if he had received a proper professional education he would feel it beneath his dignity to do such a thing; and if he found that he was not doing as well as he would like to in dentistry, he would try something else. I think there are some men who would rather starve than to deceive people by advertisements or to do imperfect work for the sake of pecuniary gain.

I cannot speak from experience in this matter of advertising in the papers, as the only instances in which I have ever done anything of the kind was in the case of removal of my office, which has only happened two or three times, and at such times I have had a card inserted in the paper for a limited time; but I can understand how there might be an advantage to the practitioner, and to the public often, in having the name of the practitioner in such places that it could be readily referred to in the case of necessity. It was only a few days ago I was asked by a gentleman if I could refer him to a special practitioner. He wanted to find an oculist and he only knew of one or two whom they told him were quacks and he was therefore afraid to go to them. Of course that is an embarrassment to the public, not knowing where to go when they wish to have special and thorough work done. At the same time, it is rather difficult to say how that list should be made up and where it should appear, as for instance, supposing all the specialists should be classified under certain headings in the directory, how would one select from that list which was the best, and what guarantee would there be that it was not simply a list of people who wanted to be known as specialists in certain lines, and yet did not possess the necessary qualifications? So it is difficult to say just what should be done, and, no doubt, it always will be, until the members of the professions can be induced to live up to the rule of doing unto others as they would that others should do to them.

*Dr. Taft.*—The case which Dr. Baker cited gave me the thought that it might have been a good thing for the young man if he had gone West. He might have received a few ideas as to the best course to pursue when he arrived there.

I have been for a good while much interested in this question of ethical advertising. I have always felt that the young man who started out in life with a good character and honest purpose, and who tried to establish himself in his profession by doing faithful and conscientious work, would build up a good practice in time. He might have to do some waiting, but he would eventually succeed. I have had considerable experience in practice-building in different localities in the past thirteen years, and I know what it means to sit down and wait for patients to come to you.

As a practitioner in the West for two years, my observation has been that competition in business and professional life is of necessity so much keener there than it is here that the views of the Western dentist upon this question of ethical advertising are quite different from those held by the Eastern dentist, and I think it might be said with truth that the question of dental ethics, and what constitutes a violation of its code, is one that disturbs our profession a thousand times more than it does the general public, East or West. I know, from experience, that people in the West, especially in the large cities, do not care a farthing in the selection of their dentist, for the ethical ideas that we try to inculcate and live up to here. They have no use for a man who hides his light under a bushel. If a man is content to sit down and not let the public know in a business-like way what his profession or calling is, they will let him severely alone. They expect him to put out a substantial sign or advertisement stating who and what he is. They want to know something about him. The "hustlers" in the professions, as in all other callings in the West, are those who get practice and business, and are the ones who get it the quickest. The progressive and intelligent dentist of the West I have always looked upon, since my acquaintance with him, as a good business man, in addition to his being a professional gentleman. He might be termed a professional business man, with certain qualifications as a dentist that are honorable as well as desirable.

It is always a pleasure to listen to Dr. Smith, for he almost invariably speaks from what I would call a common-sense, practical stand-point. I am glad to hear him speak as he has done to-night, because I have a great deal of sympathy for the young man just starting out in practice to earn an honorable living; who has had to work day and night, and who has had, perhaps, to make every sacrifice to get a professional education.

The men who have large and comfortable practices are generally the ones who frame the code of ethics, and who are also the quickest to take note of any seeming violation of it.

I have had patients say to me, "Doctor, why don't you advertise and increase your business in that way? You do not even have a sign out on your windows. There is, to be sure, a little sign at the door, which might be easily overlooked if a person were passing your office in their effort to find you. I should think you would move down town into the business district, and have a sign on your windows large enough to let people know where you are."

In discussing the question of dental ethics one day with a gentleman who looks upon the practice of dentistry from a purely business stand-point, the latter remarked, "Doctor, I would just as soon patronize a man who lets the public know his qualifications as a dentist, provided I was satisfied he could do good work, as I would the man who sat down in his office as you are doing and making no effort to let the public know who you are or what you are capable of doing, except as they gradually find it out through some one else."

I mention this merely to emphasize my belief that the public care very little for our code of ethics, or for our deliberations over what constitutes an offence against it.

I have noticed that men in other professions do not appear to consider it a violation of their code of ethics because their names are continually appearing before the public. It is *necessary and right* for a man in any profession who wishes to build up a practice to introduce himself, wherever he locates, to as many people as possible. This he can always do in a perfectly honorable and legitimate way.

Let him adopt, as has been said, the golden rule as his code of ethics, and if he conducts his practice in accordance with its precepts, we can safely allow him considerable latitude in his interpretation of the code and of what constitutes legitimate and inoffensive advertising.

*Dr. Fillebrown.*—The discussion has again brought into prominence the same old fact that medical and professional associations have their codes of ethics and that the majority of the members are violating them every day, and they will go on violating them just so long as public opinion approves of it by their patronage. The serious question is, What can be done to help it? I do not believe much can be done, except so far as you carry out the principle that has been spoken of here to-night, of educating the profession and people to a higher standard of ethics. Laws govern just so far as they are the expression of public opinion. Any law that goes beyond that is of no practical effect at all. A set of English laws which secured to the people some of the most valuable property rights, became entirely extinct by legal discussions without parliamentary action, because public opinion was against them. So far as public opinion and the professional opinion advance and agree a higher professional code of ethics will prevail. Dr. Bradley, in his closing remarks, struck the keynote to the whole situation when he said that our actions should be governed by the instincts of judgment and gentlemanly conduct. Our old American Dental Association had quite an extensive code of ethics, embracing a number of articles. It was compiled by Dr. Watts years ago, and remained as adopted until the extinction of the association, a year ago. The Southern Dental Association had the same comprehensive code, and that too has passed out of existence. When the

National Dental Association was formed there was no code of ethics adopted at all. The constitution was as much as they could at the time agree upon. Last year I had the honor of being its president, and in my remarks at the last meeting I raised the question whether we should adopt a long code similar to that which I have referred to, or a simple one that every man was expected to conduct himself as a gentleman towards his patients and fellow-practitioners. After a great deal of discussion a committee was appointed to prepare a code of ethics, and it is quite possible next year something to that effect will be adopted as a code of ethics for the Association. That is the code that is recognized in the control of our army and navy and all our national offices. We may have these fulminations on the subject from time to time, and they do just so much towards elevating our ideas as to what the standard should be, but until you educate the people and students in regard to what should be expected from professional men, you will always have what we consider violations of the code of ethics.

*Dr. Werner.*—I would like to ask wherein the general rules, which we should have for our guidance, differ from those that should govern the rest of the world, as to sociology, as to the question of commerce, or from the stand-point of Christianity,—do unto others as you would that they should do to you. As to the details of the code of ethics, we must make distinction that dentistry, as it is practised to-day, is not what it was fifty years ago; it is not as medicine was or as medicine is to-day. Every art, every manufacture, every science that has in it that which is a benefit to mankind, has brought something to our assistance in dentistry. How much machinery does for us, how much assistants of all kinds do for us, much more than can be done for the general medical practitioner. When we consider the equipment and practical knowledge and experience necessary in a dental practice to-day, we older practitioners must wonder how the young man from the dental schools is going to get a living the first few years. The most consoling thought we can find for him is the fact that we know that not more than seven-tenths of the dentistry needed to-day is done, and in that respect we are much better off than they are in a great many other fields where machinery in one hour's time will do more than fifty men can do in one day. And yet that does not mean that we have not kept pace with the improvements in other arts, as you will see when you consider how a man started in dentistry forty or fifty years ago and how the young man has to start to-day. I have no doubt the case which Dr. Baker spoke of could be duplicated many times to the knowledge of those who are present right here. Where would I start to-day if I were a stranger in Boston? We are talking here among a lot of men who have been fairly, if not more than ordinarily successful, and we do not appreciate what the young man starting out has to contend with.

Medicine is not an exact science. Surgery and our mechanical dentistry are much nearer exact sciences. We have many more specifics than there are in medicine, and few of our patients die on our hands because of inability to diagnose or cure the case. But we must hold fast to the ethical, elevated ideas of gentlemanly instincts, Christian principles, and character. We must not go to the extreme of thinking that we can advertise *ad randum,* at the same time it would be ridiculous to think that we must never allow ourselves to come before the public in any way or shape; that would be the other extreme.

*Dr. Williams.*—I would like to ask the gentleman how many teeth he has seen die?

*Dr. Werner.*—The only tooth that dies is the tooth that is pulled out.

*Dr. Briggs.*—It seems to me that there is one fault in the discussion on this subject, and that is the use of the word "advertise." Now, when we speak of advertising, we include all sorts of efforts to bring the individual to the public notice. Well, I question whether that ought to be put down as a violation of any code of ethics. It seems to me the thing that is wrong, the thing that brings the harm, is the insinuation that any one man is something or will do something that other men cannot do, and whether that is done in the public print, or whether it is said to his friends or told to his patients; however, it is done, and by whatever name you may call it, advertising or any other name, that certainly is a violation of the code of ethics. A man may insert his card in some paper, calling attention to the location of his office and the time during which he may be found there, and while it is called advertising, he does not necessarily insinuate that there are not other men in the profession who are as good as he, and so long as he does not try to impress his patients or the public in any way that he has something special, or to convey the idea that he is peculiarly endowed above his fellows by Providence, or some other way, in some particular line of work, I do not see that it can be called a violation of the code of ethics.

*Dr. Rogers.*—Surely it does not behoove me to be argumentative when I am here as your guest, but this is a free country, where a man may speak his mind freely, and, as some of you have done so this evening, you will probably not object to my doing the same. Unfortunately, my "gift of gab" is limited, and the ideas which come to me when I am listening to others are like placing a seidlitz powder in an after-dinner coffee cup,—they make their escape very quickly.

There are one or two things to which I do take exception, and I shall begin with the first speaker, Dr. Bradley. I think he is in error in his argument as to the duty that the professor owes his school. To be sure, he does owe his school a duty, a responsibility, to give to it his best efforts just as a man does to any position to which he is called, but one of the main points of my argument was that if the professor of a dental school should claim for himself the right to advance his own interests by reason of his connection with that school, then he should not deny to the student the privilege of announcing to the public that he is a graduate of that school, and that he is prepared to do dentistry in all its branches, as his diploma will certify. If there is any benefit to be derived from connection with a certain school, it seems to me the student is the one who should receive it when he is starting out to build up his practice, and if, unfortunately, when he begins to travel this hard and thorny road, he is a married man, he owes quite as much to his family as the professor does to his. When we hear of a case of a graduate of a leading dental school being obliged to move from up-town apartments to a down-town tenement, it surely is a case which excites our compassion; but it should do more than that, it should lead us to inquire into the reason why a man holding the same diploma as ourselves was not able to make a success. If that man cannot get business by following the course which has been prescribed for him, who can blame him if he adopts the methods of those who are successful?

I was greatly surprised to learn this evening that there were only about seven-tenths as many dentists as there should be; but if there are many whose experience is like the one cited here this evening, I do not see much encouragement for the other three-tenths to come in.

A man cannot hope to graduate from college and set up at once in a profitable practice. It is out of the question. He must gain the confidence of the people before he can expect patronage, and he is expected to do this without advertising himself; but if gentlemen who are in the positions of professors in schools or are connected with hospitals are allowed to call attention to these connections in the prospectus of the medical school or in the hospital reports or wherever the opportunity presents, they should not deny to the younger men some similar form of advertising.

The profession of dentistry has been spoken of to-night as the "silent profession." I was amused within a week to read in an account of the proceedings of a congress of dentists the advice given by one of the members, that dentists should cultivate a light and instructive form of conversation with which to interest the patient during dental operations.

I take exception to the statement of one of the speakers that the science of dentistry is not on the same plane with the medical profession. I do not understand what he could mean by that statement. He surely does not mean to place it on a higher plane than the medical profession, and at the same time I do not see why he should wish to place it below the medical profession and not amenable to the same laws; and if that is the case, dentists should be called by the title "dentist" and not "doctor." Surely there is a chance for brains in the work that comes before the dentists as well as in the work that comes before the medical man, and I do not see why they should not be governed by the same laws.

My whole plea is not to revolutionize the word "advertise," but to make the claim that what is "sauce for the goose is sauce for the gander," and that men who are in authority should not debar others from using the same methods for the attainment of success which they themselves employ. This distinction was brought vividly before my mind by the fact that two of my personal friends were refused membership in the New England Ophthalmological Society, because, living in New Hampshire, they had inserted in the papers a card stating their office hours, and that their practice was limited to diseases of the eye and ear. Within one week I received through the mail an address delivered by an officer of that Society. This address, which, as was stated on the envelope, was delivered by Professor ———, of Harvard University, was given a very wide circulation, and was, of course, an excellent advertisement for the gentleman. Now, that is a distinction which I claim is an injustice and is not right.

I believe heartily in what has been stated here, that the student should be educated in what is to be expected of him in the matter of ethics when he enters upon his practice, but what school is there that gives one hour a year to the instruction of its pupils on this subject? There should be a chair of ethics in every medical school, but as yet, to the best of my belief, there is no medical school which gives special instruction on this point, and the school which fails to instruct its pupils in ethics is just as much at fault as the school which would fail to instruct its pupils in the diagnosis of a cerebral tumor or how to cure a boil, and when they do send them forth without this knowledge of ethics, they should not blame them if they fall by the wayside and do a little bit of ethical advertising.

The cases reported here to-night of waiting for practice can be paralleled in the medical profession. I know of men who did not get twenty-five dollars, five dollars, or even three dollars as the result of their first year's work. The hardest case I ever heard of occurred in Schenectady, N. Y. A young man, graduate of Bellevue, told me that the first year after he opened up his office he had just two people inside,—one man to take his gas register and the other man wanted him to hire a piano, and his receipts for the first year were absolutely *nil.*

One of the speakers said that in some cases, when a man got down to a certain round of the ladder he would begin to advertise. The fact that a man does not get practice does not make it right to adopt questionable methods of advertising any more than a man who is out of work is justified in stealing. The principle is the thing at stake. We should be gentlemen first of all, but unfortunately there are some who are not gentlemen, and I have referred particularly this evening to that class who debar others from using the methods which they adopt for their own success, and while we all feel that no law can be enacted which will certainly control the actions of the members of a profession, at the same time it does no harm to have a little free conversation on the subject, and I am sure I am pleased at the full discussion it has received this evening.

*Dr. Smith.*—I do not rise to continue the argument, for I presume that the closing remarks of the essayist are supposed to close all discussion on the subject, but I wish simply to say what I think the essayist will be pleased to know, that the Dental School of Harvard University has a course of lectures for its students, wherein is taught the ethics which it is expected they shall observe in the conduct of their practice.

*Dr. Eames.*—If proper care is exercised to see that a certain standard of general education, and especially in moral ethics, is demanded of those candidates who seek to enter the colleges, then you will have a proper soil in which to implant ethical ideas.

I move that we offer a vote of thanks to our essayist for his very interesting and instructive paper.

HARRY E. CUTTER, D.D.S.,
*Editor American Academy of Dental Science.*

